# Supplementary material for: Content-rich biological network constructed by mining PubMed abstracts
Source: BMC Bioinformatics. 2004 Oct 8;5:147. doi: 10.1186/1471-2105-5-147 (PMC528731; doi:10.1186/1471-2105-5-147)
Supplement: Additional File 2 — The original results of the above study (non-essential files are deleted to keep the file size under the limit set by BMC bioinformatics). [file 1471-2105-5-147-S2.bz2 › chilibotAdditionalFile2/dip10/347ID7721786E4495/html/IL4_IL4R.html]

 


 **IL4** and **IL4R** 
  
Found 5329 abstracts in PubMed, retrieved 10.  
 

 What does Google say? 
 PDF only 
| .edu only 

---

**Interactive relationship** (e.g. stimulation, inhibition, etc)

**Non-interactive relationship** (e.g. studied together, co-existance, homology, etc.)

- A molecule solves psoriasis ? Systemic therapies for psoriasis inducing interleukin 4  [ **IL4R** ]   [ **IL4** ]  and Th2 responses.  Ref: 12879154 J Mol MedJ Mol Med,
- In vitro culture of bone marrow BM cells with granulocyte macrophage colony stimulating factor and interleukin 4  [ **IL4R** ]   [ **IL4** ]  induced a dramatic increase of telomerase activity accompanied with their differentiation into DCs.  Ref: 12885944 J Leukoc Biol, 2003
- Both Interleukin 4  [ **IL4R** ]   [ **IL4** ]  IL 4  [ **IL4** ]  and IL 4  [ **IL4** ]  Receptor alpha Signaling Contribute to the Development of Hepatic Granulomas with Optimal Antileishmanial Activity.  Ref: 12874364 Infect ImmunInfect Immun, 2003
- Interleukin 4  [ **IL4R** ]   [ **IL4** ]  levels significantly decreased P <.  Ref: 12877453 Ann Allergy Asthma Immunol, 2003
- In view of the importance of interleukin 4  [ **IL4R** ]   [ **IL4** ]  IL 4  [ **IL4** ]  gene as a genetic factor for adult periodontitis, we investigated the relationship between two polymorphisms 590 C > T polymorphism and 70 bp repeat polymorphism of the human IL 4  [ **IL4** ]  gene and adult periodontitis in the Korean population.  Ref: 12877559 Arch Pharm Res, 2003
- Neutralizing antibodies against human RANTES, MIP 1alpha, MIP 1beta, alpha interferon IFN alpha, IFN beta, IFN gamma, interleukin 4  [ **IL4R** ]   [ **IL4** ]  IL 4  [ **IL4** ] , IL 10, IL 13, IL 16, MCP 1, MCP 3, tumor necrosis factor alpha TNF alpha, or TNF beta failed to reverse the HIV 1 suppressive activity.  Ref: 12885891 J VirolJ Virol, 2003
- undetectable interleukin 4  [ **IL4R** ]   [ **IL4** ]  and interleukin 10.  Ref: 12891535 Gastroenterology, 2003
- Two polymorphisms of interleukin 4  [ **IL4R** ]   [ **IL4** ]  gene in Korean adult periodontitis.  Ref: 12877559 Arch Pharm Res, 2003
- When examined in the presence of both CD154 and interleukin 4  [ **IL4R** ]   [ **IL4** ] , Tax had a clear supportive role on B cell growth, with an impact on B cell proliferation, cell cycle phase distribution, and survival.  Ref: 12881710 Oncogene, 2003
- The roles of interleukin 4  [ **IL4R** ]   [ **IL4** ]  IL 4  [ **IL4** ]  and IL 13 in the regulation of immunity to Leishmania donovani infection are still poorly understood.  Ref: 12874364 Infect ImmunInfect Immun, 2003
- Interleukin 4  [ **IL4R** ]   [ **IL4** ]  IL 4  [ **IL4** ]  is crucial for the differentiation of naive T helper T H cells into the T H 2 effector cells that promote humoral antibody immunity and provide protection against intestinal helminths.  Ref: 12876556 Nat Rev Immunol, 2003
- Before THR, the patients showed a decrease in leukocytes and myeloid cells in comparison with healthy donors, and a prevalence of type 1 T lymphocytes, which was confirmed by the increase in ratio of interferon gamma to interleukin 4  [ **IL4R** ]   [ **IL4** ] .  Ref: 12892206 J Bone Joint Surg Br, 2003
- In the ceramic on ceramic group, leukocytes and lymphocyte subsets were not significantly changed, but a significant increase in type 2 cytokines restored the ratio of interferon gamma to interleukin 4  [ **IL4R** ]   [ **IL4** ]  to normal values.  Ref: 12892206 J Bone Joint Surg Br, 2003
